# Regenerative therapies for lumbar degenerative disc diseases: a literature review

**DOI:** 10.3389/fbioe.2024.1417600

**Published:** 2024-08-26

**Authors:** Takashi Sono, Koichiro Shima, Takayoshi Shimizu, Koichi Murata, Shuichi Matsuda, Bungo Otsuki

**Affiliations:** Department of Orthopaedic Surgery, Graduate School of Medicine, Kyoto University, Kyoto, Japan

**Keywords:** lumbar degenerative disc disease, regenerative therapy, cell therapy, biomaterials, growth factors, animal models

## Abstract

This review aimed to summarize the recent advances and challenges in the field of regenerative therapies for lumbar disc degeneration. The current first-line treatment options for symptomatic lumbar disc degeneration cannot modify the disease process or restore the normal structure, composition, and biomechanical function of the degenerated discs. Cell-based therapies tailored to facilitate intervertebral disc (IVD) regeneration have been developed to restore the IVD extracellular matrix or mitigate inflammatory conditions. Human clinical trials on Mesenchymal Stem Cells (MSCs) have reported promising outcomes exhibited by MSCs in reducing pain and improving function. Nucleus pulposus (NP) cells possess unique regenerative capacities. Biomaterials aimed at NP replacement in IVD regeneration, comprising synthetic and biological materials, aim to restore disc height and segmental stability without compromising the annulus fibrosus. Similarly, composite IVD replacements that combine various biomaterial strategies to mimic the native disc structure, including organized annulus fibrosus and NP components, have shown promise. Furthermore, preclinical studies on regenerative medicine therapies that utilize cells, biomaterials, growth factors, platelet-rich plasma (PRP), and biological agents have demonstrated their promise in repairing degenerated lumbar discs. However, these therapies are associated with significant limitations and challenges that hinder their clinical translation. Thus, further studies must be conducted to address these challenges.

## 1 Introduction

Lumbar degenerative disc disease (LDDD), an age-associated disease characterized by severe back pain and disability (Ohtori et al., 2015), is highly prevalent across the world. The pathogenesis of LDDD involves age-related (Ohnishi et al., 2018) or injury-induced (Wang et al., 2023) degeneration of the intervertebral discs (IVDs). Decreased hydration, reduced proteoglycan content, loss of disc height, annular fissuring, and ingrowth of nerve and blood vessels (Fujii et al., 2019) are observed in degenerating discs. These changes can lead to structural breakdown, biomechanical dysfunction, instability, herniation, and nerve compression. Current treatment options, such as medications (Chaparro et al., 2014), physical therapy (Hayden et al., 2021), cognitive functional therapy (Kent et al., 2023), epidural injections (Deyo and Mirza, 2016), and spinal fusion procedures (Otsuki et al., 2023; Shimizu et al., 2021), only provide temporary symptomatic relief. A treatment strategy that can restore normal disc structure, composition, and function remains to be established. Tissue engineering aims to repair and regenerate discs using cells, biomaterials, growth factors, and platelet-rich plasma (PRP). This literature review summarized the recent advances and challenges in the field of regenerative therapies for lumbar disc degeneration.

## 2 Disc structure and degeneration

**FIGURE 1:**
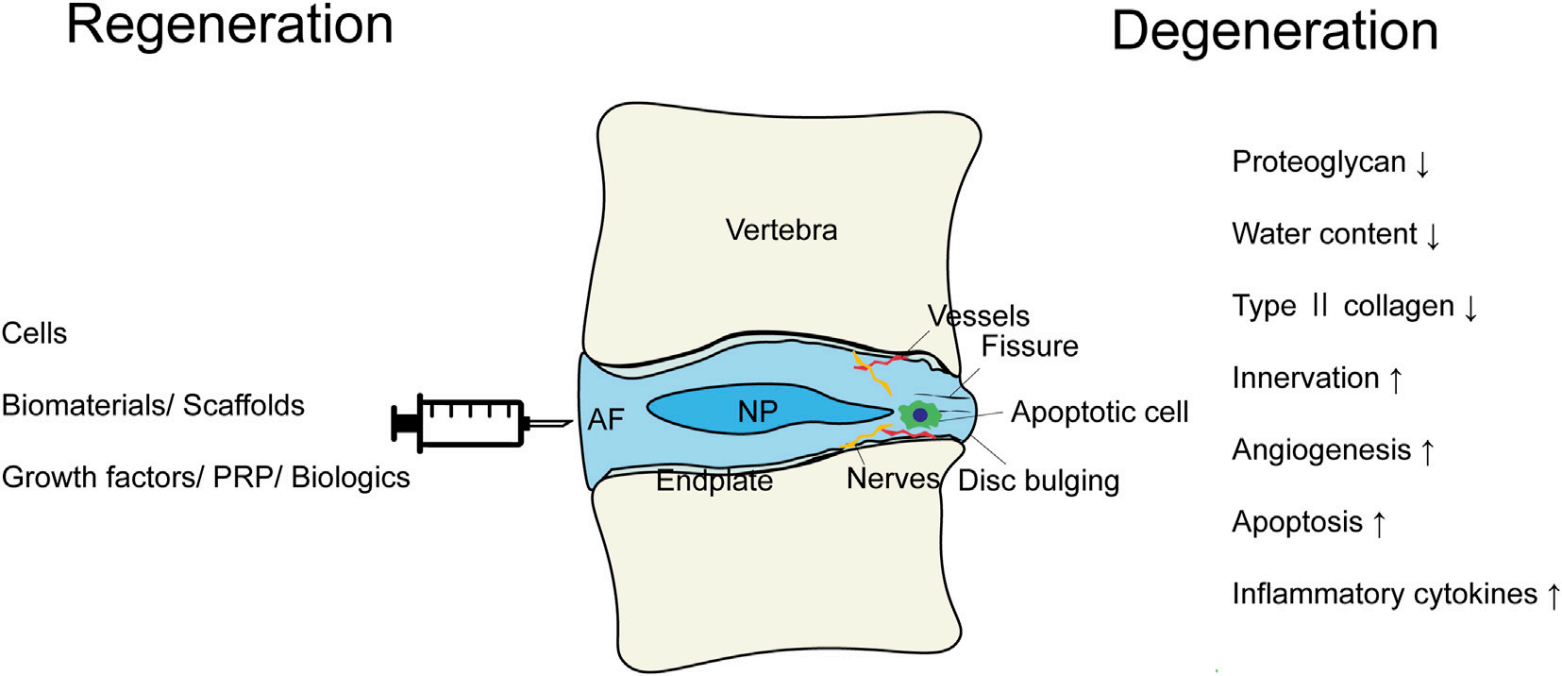
The scheme summarizing the modalities of the regenerative therapies and the pathological conditions of the intervertebral disc degeneration.

IVDs comprise a central gelatinous nucleus pulposus surrounded by the annulus fibrosus and cartilaginous endplates. The proteoglycan-rich nucleus is capable of absorbing water and resisting compressive load. The collagen fibers constituting the highly organized lamellar annulus fibrosus provide tensile strength (Sakai and Andersson, 2015). The proteoglycan and water content of the discs reduces with aging and injury. In addition, further changes, such as disorganized matrix; loss of collagen organization; ingrowth of nerves and vessels; decreased cell viability; and increased inflammatory cytokine (IL-1β, IL-6, and TNF-α), C-reactive protein levels, and type II collagen levels, have also been reported (Khan et al., 2017; de Queiroz et al., 2016; Stürmer et al., 2005; Wang et al., 2010; Goode et al., 2012) (Figure 1). These molecular changes result in structural breakdown, reduction in disc height, annular fissuring, radial bulging, altered biomechanics, nerve compression, instability, herniation, and lower back pain.

## 3 Current treatments

The first-line treatment options for symptomatic LDDD include the administration of medications, such as non-steroidal anti-inflammatory drugs, muscle relaxants, and opioids, to alleviate pain (Chaparro et al., 2014; Deyo and Mirza, 2016). Physical therapy aims to strengthen core muscles, thereby improving ergonomics (Hayden et al., 2021). Short-term pain relief can be achieved with epidural steroid injections and intradiscal electrothermal therapy (Deyo and Mirza, 2016). Spinal fusion procedures using cages and screws have been performed to stabilize the affected segments in severe cases; however, this can result in increased stress on adjacent segments (Otsuki et al., 2023; Shimizu et al., 2021). Discectomy is performed to remove herniated nucleus material compressing the nerves (Özer and Demirtaş, 2023; Goparaju et al., 2023). Disc arthroplasty involves the replacement of degenerated discs with artificial implants (van den Eerenbeemt et al., 2010; Siepe et al., 2014; Gornet et al., 2017; Park et al., 2018). Nevertheless, the inability of these approaches to modify the disease process or restore the normal structure, composition, and biomechanical function of the degenerated discs underscores the requirement for developing biological therapies that facilitate disc regeneration.

## 4 Cell sources

Cell therapy is an innovative treatment approach that involves infusing living cells into a patient to repair or replace damaged tissue or modify the behavior of local cells (Sakai and Andersson, 2015). Cell-based therapies tailored to facilitate IVD regeneration in patients with IVD degeneration have been developed to restore the IVD extracellular matrix (ECM) or mitigate the inflammatory conditions that are characteristic of disc deterioration (Fujii et al., 2019; Sakai et al., 2022). Measures should be taken to ensure that the transplanted cells thrive within the hostile environment of a degenerating IVD by directly contributing to ECM synthesis and inducing a reparative shift in native cell activity via paracrine signaling or by facilitating the arrival of regenerative cells and preventing the infiltration of harmful cells. This would help achieve successful treatment outcomes. Animal studies have demonstrated the potential of cell transplantation to decelerate or halt degenerative processes in some cases (Hiraishi et al., 2018; Nukaga et al., 2019). Minimally invasive procedures, such as a needle injection under image guidance, are used to introduce the cells into the IVD. The cells can be encapsulated in supportive matrices to enhance retention and functionality.

Mesenchymal stem cells (MSCs) have attracted considerable interest in recent years. MSCs derived from the bone marrow (Orozco et al., 2011; Elabd et al., 2016; Henriksson et al., 2019; Noriega et al., 2017; Amirdelfan et al., 2021), adipose tissue (Kumar et al., 2017), peripheral blood (Haufe and Mork, 2006), or umbilical cords (Pang et al., 2014; Lewandrowski et al., 2023) exhibit potential for chondrogenic differentiation and high proliferative ability. Furthermore, they can be harvested easily from autologous and allogeneic sources. MSCs exhibit remarkable versatility, including the capacity for immunomodulation. This property could be beneficial in tempering the inflammatory environment of the IVD (Weiss and Dahlke, 2019). Environmental stressors within the IVD can affect the proliferation and differentiation capabilities of MSCs; thus, the adaptability and survival of MSCs within IVDs require further study (Gay et al., 2019).

Human clinical trials have predominantly focused on MSCs owing to the promising outcomes exhibited by MSCs in reducing pain and improving function in patients with IVD degeneration (Table 1). Trials using adipose- and bone marrow-derived MSCs have reported improvement in pain and disability scores (Orozco et al., 2011; Elabd et al., 2016; Henriksson et al., 2019; Noriega et al., 2017; Amirdelfan et al., 2021; Kumar et al., 2017; Pang et al., 2014; Lewandrowski et al., 2023). Moreover, the safety profiles of MSCs are generally favorable, and the incidence of serious adverse events is rare (Amirdelfan et al., 2021), underscoring the potential viability of MSC therapies.

**TABLE 1:** Cell therapies for degenerative disc disease.

Author	Year	Trial	Cells	Cell number	Number of patients	Outcomes	Follow-up period (months)	Adverse events
Orozco (Orozco et al., 2011)	2011	Phase 1/2	Autologous BM-MSCs	1.0 × 10^7^	10	Significant improvement in VAS, OD,SF-36	12	None
Elabd (Elabd et al., 2016)	2016	case series	Autologous BM-MSCs	1.5 × 10^7^–5.2 × 10^7^	5	Improvement in strength and mobility	48–60	None
Henriksson (Henriksson et al., 2019)	2019	case series	Autologous BM-MSCs	1.0 × 10^6^	4	Not mentioned	8–28	One deterioration in low back pain
Noriega (Noriega et al., 2017)	2017	RCT, Phase 1/2	Allogenic BM-MSCs	2.5 × 10^7^	12	Significant improvement in VAS and OD	12	None
Amirdelfan (Amirdelfan et al., 2021)	2021	RCT, Phase 2	Allogenic BM-MSCs	6.0 × 10^6^–1.8 × 10^7^	60	Improvement in VAS and ODI	36	One implantation site infection
Kumar (Kumar et al., 2017)	2017	Phase I	Autologous AD-MSCs	2.0 × 10^7^–4.0 × 10^7^	10	Significant improvement in VAS, ODI, SF-36	12	None
Haufe (Haufe and Mork, 2006)	2006	case series	Autologous HSCs	NA	10	No improvement in pain	12	None
Pang (Pang et al., 2014)	2014	case series	Allogenic UC-MSCs	1.0 × 10^7^	2	Improvement in VAS and ODI	24	None
Lewandrowski (Lewandrowski et al., 2023)	2023	case series	Allogenic UC-MSCs	5.0 × 10^6^	33	Significant improvement in VAS and ODI	24	None
Mochida (Mochida et al., 2015)	2015	case series	Autologous NP cells	1.0 × 10^6^	9	Improvement in JOA score and lumbar back pain	36	None
Beall (Beall et al., 2021)	2021	RCT	Allogenic spine-derived cells	>6.0 × 10^6^	123	Significant improvement in ODI and VAS	12	2/141 SAEs
Coric (Coric et al., 2013)	2013	Phase 1	Allogenic chondrocytes	1.0 × 10^7^–2.0 × 10^7^	15	Significant improvement in NRS, ODI, and SF-36	12	None
Tschugg (Tschugg et al., 2017)	2017	RCT, Phase 1/2	Autologous disc-derived chondrocytes	3.6 × 10^6^–4.4 × 10^6^	12	Not mentioned	1.5	6/12 TEAEs
Comella (Comella et al., 2017)	2017	case series	Autologous SVF cells/PRP	3.0 × 10^7^–6.0 × 10^7^	15	Significant improvement in VAS, PPI, and SF-12	12	None
Pettine (Pettine et al., 2015)	2015	case series	Autologous BMC	1.2 × 10^7^	26	Significant improvement in ODI, VAS	12	None
Tuakli-Wosornu (Tuakli-Wosornu et al., 2016)	2016	RCT	PRP	NA	29	Significant improvement in NRS and function	12	None
Akeda (Akeda et al., 2022)	2022	RCT	PRP	NA	9	Significant improvement in RDQ and JOABPEQ	60	One post-injection pain

BM, bone marrow-derived; MSC, mesenchymal stem cell; VAS, visual analog scale; ODI, oswestry disability index; SF, Short Form. RCT, randomized controlled trial; AD, adipose-derived; HSC, hematopoietic precursor stem cell; UC, umbilical cord-derived; NP, nucleus pulposus. JOA, japanese orthopedic association; SAEs, serious adverse events; TEAEs, treatment-related adverse events; SVF, stromal vascular fraction. PRP, platelet-rich plasma; PPI, present pain index; BMC, bone marrow concentrate; NRS, numeric rating scale; RDQ, roland morris disability questionnaire. BPEQ, back pain evaluation questionnaire.

Studies on other types of cells, such as the nucleus pulposus (NP) cells (Mochida et al., 2015; Beall et al., 2021) and chondrocytes from articular or hyaline cartilage (Coric et al., 2013; Tschugg et al., 2017), have elucidated the unique regenerative capacities inherent to cells native to avascular tissues, such as the IVD. However, the limitations associated with accessibility and phenotypic stability limit the widespread application of these treatment strategies. Clinical application of these cells, although limited, suggests their safety and potential efficacy in improving the status of the IVD and patient outcomes.

The treatment outcomes of emerging therapies utilizing less-defined cell products, such as stromal vascular fraction (SVF) (Comella et al., 2017) and bone marrow concentrate (BMC) (Pettine et al., 2015), vary. These complex mixtures, which comprise various types of cells and bioactive factors, offer a multifaceted approach to tissue regeneration. The precise mechanisms of action and individual contributions of these components are unclear; however, early results suggest a potential for facilitating significant clinical improvement in certain cases (Comella et al., 2017; Pettine et al., 2015). Nevertheless, the heterogeneity of these products hinders standardization and quality control.

Animal experiments have used iPS cells as a cell source and induced differentiation into notochordal cells (Sheyn et al., 2019), nucleus pulposus-like cells (Zhang et al., 2020), or cartilaginous tissue (Kamatani et al., 2022). These cells were transplanted into IVDs subsequently. Their application in human beings is anticipated in the future.

## 5 Biomaterials and scaffolds

Biomaterials aimed at NP replacement in IVD regeneration, comprising synthetic and biological materials designed to restore disc height and segmental stability without compromising the annulus fibrosus, are predominantly injectable materials (Iatridis et al., 2013; Mehrkens et al., 2012). The properties of synthetic materials must match the mechanical properties of the native disc to achieve successful outcomes. This ensures the integration of the material with the surrounding structures and the restoration of the motion characteristics without inducing adverse immune responses. Furthermore, durability and minimal wear-debris generation are critical factors affecting clinical viability (Bowles and Setton, 2017; Li et al., 2022).

Biologically based materials can be remodeled by the body. Consequently, a different set of criteria, primarily focusing on the ability to support cell-mediated tissue regeneration, must be satisfied by these materials to achieve successful outcomes. This category comprises materials that serve as cell-delivery vehicles to promote NP tissue regeneration (Bowles and Setton, 2017; Li et al., 2022).

Initial strategies for NP replacement involved the administration of synthetic polymers that hydrate *in situ* to mimic the natural hydration of NP. This approach aimed to restore disc pressure and height. Copolymeric hydrogel (polyacrylonitrile [PAN] and polyacrylamide) encased in a polyethylene jacket (PDNTM) is one such combination (Ray, 2002). Other materials, such as polyvinyl alcohol (PVA) and polyvinyl pyrrolidone (PVP) reinforced with a Dacron mesh (NeuDisc™) (Bertagnoli et al., 2005),hydrating PAN (NucleoFix™^/^Gelstix™) (Bowles and Setton, 2017; Ceylan et al., 2019; Koetsier et al., 2022), *in situ* curing polymer using inflatable polyurethane balloon (DASCOR™) (Tsantrizos et al., 2008; Ahrens et al., 2009), *in situ* curing polymerized water-in-oil emulsion composite (DiscCell™) (Pelletier et al., 2016), glutaraldehyde cross-linked elastin and silk polypeptide (BioDisc™) (Yuksel et al., 2002), and hydrogel of a chemically cross-linked elastin and silk polypeptide (NuCore™) (Berlemann and Schwarzenbach, 2009) have also been used. However, these materials are associated with significant limitations such as uncontrolled swelling, mechanical complications (e.g., stiffness), and device migration. Clinical trials have explored various materials, such as hydrogels and polymers (Ceylan et al., 2019; Koetsier et al., 2022; Ahrens et al., 2009; Berlemann and Schwarzenbach, 2009), that can transition into a gel or solid state *in situ*, to minimize the damage to the annulus fibrosus during implantation. Ceylan et al. (2019) reported GelStixTM demonstrated mean VAS score and ODI score improvement after implantation. Moreover, RCT is being conducted (NCT 02763956) (Koetsier et al., 2022). However, progress in the domain of market approval has been limited (Bowles and Setton, 2017) (Table 2) owing to the focus on mechanical restoration rather than biological integration or interaction with local cells.

**TABLE 2:** Clinical and preclinical results of the nucleus pulposus repair or replacement devices.

Trade name	Classification	Polymer	Outcomes
NeuDisc™ (Bertagnoli et al., 2005)	injectable	Copolymer of polyvinyl alcohol and polyvinyl pyrrolidine or modified PAN reinforced by a Dacron mesh	Force to failure 3581N in compression
NucleoFix™ /GelStix™ (Bowles and Setton, 2017) (Ceylan et al., 2019) (Koetsier et al., 2022)	injectable	Hydrating PAN	Mean VAS score and ODI score were improved significantly RCT is being conducted (NCT02763956)
DASCOR™ (Tsantrizos et al., 2008) (Ahrens et al., 2009)	*in situ* forming	*in situ* curing polymer using inflatable polyurethane balloon	4.2–5.6 MPa compressive strength Significant improvements in mean ODI and VAS scores
DiscCell™ (Pelletier et al., 2016)	*in situ* forming	*in situ* curing polymerized water-in-oil emulsion composite	Restoring segmental range of motion in vitro
BioDisc™ (Yuksel et al., 2002)	*in situ* forming	Glutaraldehyde cross-linked elastin and silk polypeptide	Mechanical durability of the implant was demonstrated through 10 million compressive loading cycles
NuCore™ (Berlemann and Schwarzenbach, 2009)	*in situ* forming	Hydrogel of a chemically cross-linked elastin and silk polypeptide	Significant improvement for leg and back pain, as well as function scores

PAN, polyacrylonitrile; VAS, visual analog scale; ODI, oswestry disability index; RCT, randomized controlled trial.

Engineering complete IVD replacements using materials that facilitate cellular survival and matrix remodeling has garnered interest in recent years, indicating a trend toward the use of more biologically integrated solutions. Composite IVD replacements that combine various biomaterial strategies to mimic the native disc structure, including organized annulus fibrosus and NP components, have shown promise (Mizuno et al., 2006; Sakai et al., 2006; Nesti et al., 2008; Nerurkar et al., 2010; Zhuang et al., 2011; Bowles et al., 2011; Park et al., 2012; Martin et al., 2014; Choy and Chan, 2015; Chik et al., 2015; Ukeba et al., 2021) (Table 3). This strategy aims to replicate the appearance and mechanical functions of the native disc; however, it has exhibited varying degrees of success in mimicking the mechanical properties and achieving integration with the native tissue.

**TABLE 3:** Overview of the composite materials with cells.

Author	Year	Materials	Donor cells	Recipients	Outcomes
Mizuno (Mizuno et al., 2006)	2006	PGA/alginate gel	sheep AF cells/NP cells	mice	Proteoglycan, collagen content and compressive mechanical properties were similar to native NP cells
Sakai (Sakai et al., 2006)	2006	atelocollagen	rabbit bone marrow derived MSCs	rabbit	Regained disc height, proteoglycan accumulation and T2-weighted signal intensity in MRI
Nesti (Nesti et al., 2008)	2008	PLLA/HA	human bone marrow derived MSCs	NA	The composite IVD expressed type 1,2,9,10,11 collagen and aggrecan
Nerurkar (Nerurkar et al., 2020)	2010	electrospun PCL/agarose	bovine AF cells/bone derived MSCs	NA	Appropriate ECM deposition was found in the composite IVD
Zhuang (Zhuang et al., 2011)	2011	DBM/collagen2/hyaluronate/chondroitin-6-sulfate	rabbit AF cells/NP cells	mice	Collagen and proteoglycan deposition was found in the composite IVD
Bowles (Bowles et al., 2011)	2011	contracted collagen gel/alginate	ovine AF cells/NP cells	rat	Disc height was maintained in half of the implants
Park (Perk et al., 2012)	2012	porous silk/fibrin/HA hydrogel	porchine AF cells/chondrocytes	NA	Implanted AF cells and chondrocytes demonstrated appropriate gene expression and GAG especially in the lamellar silk scaffold
Martin (Martin et al., 2014)	2014	electrospun PCL	None	rat	Construct was stable in 47% of samples without external fixation and cell infiltration into implants was found
Choy (Choy and Chan, 2015)	2015	photochemically crosslinked collagen membranes/collagen-GAGs co-precipitate	None	NA	Composite IVD showed as good performance as the native disc on mechanical testing
Chik (Chika et al., 2015)	2015	contracted collagen gel, collagen-GAGs co-precipitate	rabbit bone marrow derived MSCs	NA	Engineered IVD showed appropriate histological features
Ukeba (Ukeba et al., 2021)	2021	ultra-purified alginate gel	bone marrow derived stem cell/bone marrow aspirate concentrate	rabbit	Composite IVD demonstrated good mechanical properties and enhanced repair of IVD defects in rabbits

PGA, polyglycolic acid; AF, annulus fibrosus; NP, nucleus pulposus; MRI, magnetic resonance image; ECM, extracellular matrix; IVD, intervertebral disc; PLLA, poly L-lactic acid; HA, hyaluronic acid; NA, not assessed; DBM, demineralized bone matrix. MSC, mesenchymal stem cell; GAG, glycosaminoglycan; PCL, poly ε-caprolactone.

Challenges related to the management of the disc space, integration with the native tissue, and the mechanical environment of the spine have been reported by *in vivo* animal studies on composite discs. These findings emphasize the requirement for conducting further research to address these limitations that hinder successful clinical translation (Mizuno et al., 2006; Sakai et al., 2006; Zhuang et al., 2011; Bowles et al., 2011; Martin et al., 2014; Ukeba et al., 2021). Techniques to engineer motion segments, including the disc and adjacent bony structures, aim to overcome the challenges associated with integration and improve patient outcomes.

Although promising, the field of IVD regeneration through NP replacement is associated with several limitations associated with material design, biological integration, and mechanical performance. Thus, further studies must be conducted in the future to better understand and overcome these obstacles for successful clinical application.

## 6 Growth factors, PRP, and biologics

Growth factors, such as TGF-β (Sun et al., 2023; Risbud et al., 2006); GDF-5,6 (Gantenbein-Ritter et al., 2011; Clarke et al., 2014); FGF-2 (Tsai et al., 2007); IGF-1 (Osada et al., 1996); BMP-2, 4, and 7 (Tim et al., 2003; Du et al., 2022; Ellman et al., 2013); and CTGF (Matta et al., 2018), have been evaluated in previous studies (Table 4) These factors can stimulate matrix synthesis, cell proliferation, and differentiation. However, concerns regarding spatiotemporal delivery and uncontrolled differentiation remain.

**TABLE 4:** Growth factors associated with intervertebral disc regeneration.

Author	Year	Name of growth factor	Dose	Animals	Outcomes
Sun (Sun et al., 2023)	2023	TGF-β1	Plasmid	Human NP cells	Modulating oxidative stress
Risbud (Risbud et al., 2006)	2006	TGF-β3	10 ng/mL	Rat NP and AF cells	Maintenance of phenotype
Gantenbein Ritter (Gantenbein-Ritter et al., 2011)	2011	GDF-5	100 ng/mL	Human BMSCs	Upregulation of Col2, ACAN
Clarke (Clarke et al., 2014)	2014	GDF-6	100 ng/mL	AD-MSCs, BM-MSCs	Upregulation of Col2, ACAN and NP marker
Tsai (Tsai et al., 2007)	2007	FGF-2	10 ng/mL	Bovine NP cells	Maintenance of phenotype
Osada (Osada et al., 1996)	1996	IGF-1	100 ng/mL	Bovine NP cells	Proteoglycan synthesis
Yoon (Tim et al., 2003)	2003	BMP-2	1000 ng/mL	Rat AF cells	Upregulation of Col2, ACAN and Sox9
Du (Du et al., 2022)	2022	BMP-4	68 ng/mL	Sheep NP and AF cells	Upregulation of Sox9 and increased ECM production
Ellman (Ellman et al., 2013)	2013	BMP-7	100 ng/mL	Bovine NP cells	PG synthesis and upregulation of ACAN
Matta (Matta et al., 2018)	2018	CTGF	100 ng/mL	Human NP cells, rat, dog	Suppression of inflammation and upregulation of Col2, ACAN

TGF, transforming growth factor; NP, nucleus pulposus; AF, annulus fibrosus; GDF, growth differentiation factor. BMSC, bone marrow-derived stem cell; Col2, type II collagen; ACAN, aggrecan; AD, adipose-derived. MSC, mesenchymal stem cell; BM, bone marrow-derived; FGF, fibroblast growth factor; IGF, insulin-like growth factor. BMP, bone morphogenetic protein; Sox9, SRY-Box Transcription Factor 9; ECM, extracellular matrix; PG, proteoglycan. CTGF, connective tissue growth factor.

Injecting GDF-6 into the IVD attenuated inflammatory gene expression and improved disc degeneration in a rabbit puncture model (Miyazaki et al., 2018). Phase 1/2 clinical trials on the intradiscal injection of recombinant human growth and differentiation factor-5 (rhGDF-5) have reported promising results (NCT00813813 and NCT01124006).

PRP, which comprises concentrated autologous platelets and growth factors (Akeda et al., 2019), has shown potential in increasing cell proliferation, matrix production, and disc height in preclinical models. PRP can be classified into four categories, comprising four types of preparation, based on the number of leukocytes and fibrin content (Akeda et al., 2019). Two randomized controlled trials have investigated the effects of PRP. Tuakli-Wosornu et al. reported that injecting PRP into the IVD resulted in significant improvement in lower back pain and function over 8 weeks; moreover, the improvement was maintained at the 1-year follow-up visit (Tuakli-Wosornu et al., 2016). Akeda et al. reported that injecting PRP into the IVD resulted in a significant improvement in the disability score at 26 weeks and walking ability scores at 4 and 8 weeks compared with those achieved with corticosteroid injection (Akeda et al., 2022) (Table 1). Optimal formulations and delivery methods continue to be explored. Small molecules and drugs aim to inhibit inflammatory cytokines and MMPs, while also regulating the expression of catabolic/anabolic genes.

Nuclear factor-κB (NF-κB) decoy and TNF-α inhibitors are biologics that exert anti-inflammatory effects. NF-κB, a transcription factor, regulates the inflammatory cytokine levels. NF-κB decoy, an oligodeoxynucleotide containing the NF-κB binding site that entraps NF-κB subunits, can suppress NF-κB activity. Intradiscal injection of NF-κB can suppress inflammatory gene expression in degenerated discs and restore disc height loss (Kato et al., 2021). Notably, intradiscal injection of a TNF-α inhibitor (etanercept) improved discogenic pain in humans within a 2-month follow-up period in a previous study (Sainoh et al., 2016).

## 7 Multi-strategy synergistic therapy

In recent years, studies on the multi-strategy synergistic therapies that incorporate cells, biomaterials, and growth factors for intervertebral disc degeneration have also been conducted. Wei et al. reported that TGF-1 was embedded with MSCs in decellularized annulus fibrosus matrix (DAFM) hydrogels reinforced with polyethylene glycol diacrylate (PEGDA) to facilitate the controlled release of TGF-1 while preserving the hydrogel’s porous structure. Following infusion into a rat model with AF injury, there was a notable increase in the migration of AF cells to the injury site, which promoted anabolic upregulation (Wei et al., 2022). Sun et al. (2020) utilized 3D printing and electrospinning technology to load TGF-β3, CTGF and bone marrow-derived MSCs onto polydopamine nanoparticles and polycaprolactone scaffolds, respectively, mimicking the structure of AF and achieving mechanical properties similar to those of natural AF in the rodents. Although these studies have demonstrated that multi-strategy combination has great potential for IVD repair, further studies in large animal models are necessary.

## 8 Animal models

*In vitro* and *in vivo* studies have been conducted using small and large animal models of rodents (such as mice and rats), rabbits; dogs; sheep; goats; pigs; and monkeys; to evaluate regenerative techniques (Poletto et al., 2023). The use of small animal models is a cost-effective approach for screening. Rodents, such as mice and rats, are the most commonly used animal species (54%) in IVD studies (Poletto et al., 2023). However, the physiological characteristics of rodents vary from those of humans. In contrast, the disc size and disc degeneration observed in large animals, such as dogs, sheep, and pigs, is similar to that in humans; nevertheless, replicating the slow, progressive human disc pathology in these animals is difficult. IVD degeneration can be induced via bacterial, chemical, genetic, noninvasive, spontaneous, and surgical methods (Poletto et al., 2023; Oichi et al., 2020). The experimental time points vary depending on the species. The commonly used time points for different species are as follows: rodents, 2 or 4 weeks; rabbits, 4 weeks; sheep, 24 weeks; goats, 12 weeks; pigs, 12 weeks; and monkeys, >104 weeks (Poletto et al., 2023). A single animal model that can recapitulate the entirety of human IVD degeneration remains to be established. However, the results can be interpreted reliably if the limitations of a selected animal species are recognized.

## 9 Clinical translation

Preclinical studies have demonstrated the promising results of regenerative disc therapies. Nevertheless, these therapies are associated with significant limitations that hinder their translation into clinical practice. These limitations include insufficient graft retention and integration into the disc space, limited survival and proliferation of the cells within the harsh disc microenvironment, uncontrolled differentiation of stem cells, mechanical instability of the implanted scaffolds, and concerns regarding long-term safety. Furthermore, the regulatory requirements for devices and biologics set forth by the U.S. Food and Drug Administration also pose hurdles. Clinical implementation of regenerative disc therapies requires optimization of cell sources, biomaterials, growth factors, PRP, gene therapy, mechanical stimulation, and delivery methods. Evaluation of clinically relevant models and personalized regenerative therapies tailored to individual patients can be achieved using biomarkers, advanced imaging modalities, and bioreactors.

## 10 Discussion

Preclinical studies on regenerative medicine therapies that utilize cells, biomaterials, growth factors, PRP, and biological agents have demonstrated their promise in repairing degenerated lumbar discs. However, these therapies are associated with significant limitations and challenges that hinder their clinical translation. Thus, further studies must be conducted in the future to address these challenges. Advances in the fields of tissue engineering, biomaterials, stem cells, and biological factors will facilitate regenerative therapies to halt or reverse progressive disc degeneration and improve the clinical outcomes and quality of life of patients with LDDD.
